# A 90-Day Oral Toxicity Study of the Ethanol Extract from *Eupatorium japonicum Thunb* and *Foeniculum vulgare* in Rats

**DOI:** 10.1155/2020/6859374

**Published:** 2020-06-27

**Authors:** Guangcheng Dai, Chenglu Wang, Wei Tang, Jiangyun Liu, Boxin Xue

**Affiliations:** ^1^Department of Urology, The Second Affiliated Hospital of Soochow University, Suzhou 215004, China; ^2^Department of Reproductive Endocrinology, Zhejiang Provincial People's Hospital, Hangzhou Medical College, Hangzhou 310014, China; ^3^College of Pharmaceutical Sciences, Soochow University, Suzhou 215123, China

## Abstract

*Eupatorium japonicum Thunb* and *Foeniculum vulgare* are two of the most widely used folk herbs and constituents in many traditional Chinese herbal formulas. Nonetheless, little toxicological and safety information associated with following daily repeated exposure is obtained according to previous research. The present study was performed to assess the toxicity of ethanol extract from *Eupatorium japonicum Thunb* and *Foeniculum vulgare* (EFE) in male rats administered by dietary oral gavage at target doses of 0.39, 0.78, and 1.56 g/kg body weight/day for 90 days. There were no significant adverse effects on clinical signs, body weight, food conversion efficiency, and vital hematological indices. However, some hematology and biochemical indices such as WCV, MCH, MCHC, LY, MPV, T-CHO, as well as TG revealed significant changes in Sprague–Dawley rats and organ weights in lung and spleen showed diminished in male rats. Necropsy and histopathology findings suggested that no significant differences in absolute weights were found in all organs except lung and spleen, and no treatment-related alteration was identified in any organs. All results obtained in the present study indicated that the proper use of EFE in traditional medicine at oral dosages up to 1.56 g/kg/day body weight may harbor no prolonged toxicity to rats. However, further studies of EFE are still necessary to assess its oral safety in patients.

## 1. Introduction

*Eupatorium japonicum Thunb* and *Foeniculum vulgare* are widely used by folk and traditional medicines in many ancient Chinese herbal formulas. *Eupatorium japonicum Thunb*, belonged to the family of *Asteraceae* in the *Asterales* within the Angiosperms, is widespread in China, Japan, and Korea. According to previous researches, the leaves and stems have been shown the effect of anti-inflammatory and vascular smooth muscle relaxant (1). Therefore, *Eupatorium japonicum Thunb* are identified as the effective antivirals, antibacterials, diuretics, painkillers, vermifuges, and carminatives (2). And it is utilized for the treatment of vomiting, indigestion, nausea, and diarrhea (The Chinese People's Pharmacopoeia, 2005) (3). *Foeniculum vulgare*, the oldest valid name within the genus *Foeniculum*, is a flowering plant species widely cultivated in almost all countries of the world (4). It is an ancient and popular herb with a long utilization history. Several past studies have shown that numerous infections disorders including bacterium, fungus, virus, and mycobacterium can be effectively controlled using *Foeniculum vulgare* (5–7). It has been suggested that *Foeniculum vulgare* has multiple biological functions such as cytoprotective, hypoglycemic, chemopreventive, hepatoprotective, and oestrogenic activities according to previous literature (8–12). Recently, some researchers stated that *Foeniculum vulgare* can be used for management stress-related disorders because of its antistress and memory-enhancing function (13).

In our previous study, Ethanol extract from *Eupatorium japonicum Thunb* and *Foeniculum vulgare* (EFE) can regulate the balance of sex hormone and decrease the hyperplasia epithelia of prostate in male Sprague–Dawley (SD) rats, which might result in an inhibitory effect of benign prostatic hyperplasia (14). Nonetheless, to our knowledge, no data are available so far on the safety assessment of EFE in animals. Therefore, the present research investigated the potential toxicity of EFE using a 90-day feeding study in male SD rats.

## 2. Materials and Methods

### 2.1. Materials

#### 2.1.1. Preparation of EFE

The whole plants of *Eupatorium japonicum* Thunb and *Foeniculum vulgare* were purchased from the local Chinese herb market and the voucher specimens were deposited in the herbarium of College of Pharmaceutical Sciences, Soochow University (Suzhou, China).

For the ethanol extract preparation, formula herbs 20 kg (*Eupatorium japonicum* Thunb. and *Foeniculum vulgare were marked, respectively*, 1 : 1), dried and teared for preparation, reflux extraction twice with 10 times 85% ethanol, 1.5 hours every time, merge extracting solution, decompression enrichment to a quarter of the volume and let stand for the night, precipitation with Buchner funnel, filtration solution concentration under vacuum to the density of 1.05 in the temp of 60°C, the ethanol solution is concentrated by reducing pressure and dried by vacuum; finally, 1.56 kg block solid extract is obtained, which was utilized in the following tests. The yield of EFE was 7.8%.

### 2.2. Experimental Animals

Forty male adult Sprague–Dawley rats, 6 weeks old, 124.5-142.0 g, were purchased from the Slac Laboratory Animal Co., Ltd. (Shanghai, China). Mice were individually kept in cages with stainless steel grid covers, bedded with sterilized wood shavings, and were cared in a controlled environment. The commercial diets (Suzhou Shuangshi Laboratory Animal Feed Science and Technology Co., Ltd. Soochow) and tap water were provided *ad libitum*. The rats were allowed 1 week for acclimation prior to the conduction of experiments.

All animals received human care according to the Chinese legislation on the usage and care of laboratory animals. The study was approved by the committee of Soochow University of Science and Technology.

### 2.3. Study Design Overview

The study was performed based on the Procedure and Methods of Food Safety Toxicological Assessment, GB15193.13-2003 (in Chinese) (15).

#### 2.3.1. 90-Day Period of Feeding Study

After getting 7-day periods of acclimatization, forty rats were used in the study. The dose was discussed according to the results of previous and pharmacodynamic and acute oral toxicity study. All rats were randomly selected and included into three treatment groups and a control group. The oral feeding was continually conducted (i. g.) at the same time from Monday to Saturday each week, during the 90-day period for EFE-1(0.39 g/kg/day), EFE-2 (0.78 g/kg/day), EFE-3 (1.56 g/kg/day), and control (water, 10 ml/kg/day) groups (14).

Signs of toxicity and mortality were observed throughout the experimental period. All rats were weighed at the beginning of the administration, then recorded every four days during the experimental period and at the end of the study. Also, food consumption was calculated every four days, and total food consumption for each rat was assessed by summation of the food intake recorded from every feeding period. Furthermore, food conversion efficiency was measured as the weight gain per gram of food consumed.

At the end of the 90-day oral feeding, rats were sacrificed after diets withdrawing 16 h. Blood from abdominal aorta were collected and divided into two tubes for blood hematological and chemistry analysis, respectively. We then weighed and performed histopathological examinations on organs such as heart, spleen, lung, liver, kidney, brain, thymus, adrenal gland, testes, and epididymis.

### 2.4. Observations

#### 2.4.1. Clinical Signs

We continuously monitored the changes every day in physical appearance, behavioral pattern, breath, glandular secretion, excreta, mortality, and signs of illness throughout the experiment. Additionally, the body weight of each rat was observed every four days in our oral toxicity study.

#### 2.4.2. Body Weight, Food Consumption, and Food Conversion Efficiency

Body weight was recorded on the first day of administration, then recorded every four days during the 90-day feeding and at the end of the experiment. Food consumption was evaluated over successive periods of 90 days by weighing the rats. In addition, food conversion efficiency was measured as the weight gain per gram of food consumed.

#### 2.4.3. Hematological and Biochemical Analyses

Hematological parameters, including white blood cell (WBC), red blood cell (RBC), hemoglobin (Hb), five-classification of WBC (Neutrophil, NE/Lymphocyte, LY/Eosinophil, EO/Monocyte, MO/Basophil, BA), hematocrit (HCT), mean corpuscular hemoglobin (MCH), mean corpuscular volume (MCV), mean corpuscular hemoglobin concentration (MCHC), mean platelet volume (MPV), and red cell distribution width-coefficient of variation (RDW-CV), was analyzed by automatic hematology analyzers XE2100 (Sysmex, Japan). Biochemical parameters such as alanine aminotransferase (ALT), total protein (TP), aspartate aminotransferase (AST), albumin (ALB), blood urea nitrogen (BUN), creatinine (CERA), triglyceride (TG), and total cholesterol (T-CHO) were measured by Cobas 8000 Biochemistry Automatic Analyzer (Roche, Germany).

#### 2.4.4. Organs Weight and Histopathological Examinations

At the ending of the experiment, all the rats were euthanized and subjected to necropsy. Pertinent observations on the pathology of each organ were conducted and recorded. Organs such as heart, lung, liver, spleen, kidney, brain, thymus, adrenal gland, testes, and epididymis were weighed before the histopathological tests. Histopathological procedures were conducted according to standard protocols. After trimming at 2-3 mm thickness, formalin-fixed tissues were embedded in paraffin. Moreover, the tissues were cut at sections in 5 *μ*m thick and stained with haematoxylin and eosin (H&E). Microscopic examination was performed on the major organs in all rats at the 1.56 g/kg/day dosage treatment groups. The findings were compared to parallel sections at 0.39 g/kg/day and 0.78 g/kg/day dosage group as well as the control group.

### 2.5. Statistical Analysis

All values were presented using means ± SD. The differences between the control and dosage groups were statistically evaluated by ANOVA. *P* value < 0.05 was considered to be statistically significant. Statistical analyses were conducted by SPSS 24.0 software.

## 3. Results

### 3.1. Clinical Observations

All the experimental animals survived and appeared healthy during the 90-day period of the study. No treatment-related changes were discovered in daily general observations and clinical examinations.

### 3.2. Body Weight, Food Consumption, and Food Conversion Efficiency

After the 90-day oral feeding, no statistically significant changes were found in body weights, food consumed, and food conversion efficiency between the treatment and control groups (Table 1).

### 3.3. Hematology and Biochemical Data

The hematological data were presented in Table 2. No significant changes of hematological parameters were found between the EFE-2 and control group. As for the EFE-1 and EFE-3 group, vital hematological indices including WCV, MCH, MCHC, LY, and MPV were significantly changed (*P* < 0.05) compared to control.

The serum biochemical parameters were summarized in Table 3. In comparison with the control group, no statistically significant differences were discovered in the EFE-1 and EFE-2 group. In comparison to the controls, T-CHO and TG contents of the EFE-3 group had a significant decrease (*P* < 0.05).

### 3.4. Organ Weights and Pathology

Except for lung and spleen, no significant differences in absolute organ weights were discovered in the EFE groups compared with controls (Table 4). The clinical and pathologic examinations in organs indicated that no significant change was detected between treatment and control groups (data not shown).

## 4. Discussion

*Eupatorium japonicum Thunb* and *Foeniculum vulgare* are widely used in many Asian countries and are considered as the major active components in lots of classic traditional Chinese medicine herbal formulas. Previously, the extracts of *Eupatorium japonicum Thunb* were found to contain the essential oil thymol and several pyrrolizidine alkaloids such as rinderine, amabiline, echinatine, indicine, and viridiflorine (16). Gu et al. (1) reported that ethanol extract of the flowers was one of the suppressors of nitric oxide synthase expression. They conducted a further study of the mechanism of *Eupatorium japonicum Thunb* for anti-inflammatory effects. Their results provide that it can regulate the Toll/interleukin 1 receptor domain-containing adapter inducing interferon-*β*-dependent pathways, which lead to the decrease of inflammatory gene expression (17). *Foeniculum vulgare* has been extensively utilized in traditional medicine for the treatment of a wide range of diseases. Although the previous studies indicated estragole genotoxicity and hepatocarcinogenicity of *Foeniculum vulgare* in mice, no DNA damage caused by this medical plant was found in human hepatic cells (18–21). The available scientific study has suggested that it is a critical medical plant for the management of arthritis, cancer, diarrhea, constipation, fever, gastritis, and stomachache (22–30). According to a previous report, Shah and colleagues conducted the toxicity study of the ethanolic extract of *Foeniculum vulgare* in male and female mice. 3 doses (0.5, 1, and 3 g/kg) for acute toxicity and 100 mg/kg for the 90-day toxicity dose were used in that research. They concluded that the extracts caused no significant acute or chronic mortality to mice (31). In another study, the juice of the aerial parts of this species was found toxic to rats in a dose of 9.772 mg/kg/BW. In other words, *Foeniculum vulgare* leaves juice was suggested slightly toxic to rats (21). Additionally, Rather (32) reported that estragole, one of the major phytoconstituents of *Foeniculum vulgare*, was associated with the development of malignant tumors in mice. In our recent study, the traditional Chinese herbal medicine compound of *Eupatorium japonicum Thunb* and *Foeniculum vulgare* extraction (EFE) has a significant inhibitory effect on benign prostatic hyperplasia. The potential mechanism was suggested that EFE can reduce the expression of bFGF and VEGF protein while enhancing the expression of TGF-*β*protein. Moreover, the main components of EFE will be detected to determine the possible mechanism in our further study. It is well known that toxicological screening and safety evaluation on medical plants is essential before therapeutic applications in disorders and diseases. During the last several decades, acute and long-term oral feeding has been advocated as a fundamental test and applied in many safety assessing studies. Therefore, we firstly tested the EFE acute toxicity potency in ICR mice, and the results revealed that the EFE has no toxicity to ICR mice (data not shown). The present study, followed by previous toxicity study, provided evidences of EFE safety using a 90-days oral feeding with dosing up to 1.56 g/kg body weight. All toxicity tests in the present study are conducted in compliance with the guidelines of Procedure and Methods of Food Safety Toxicological Assessment (33).

During the course of the experiment, rats in all EFE groups and the control group were all survived in good health. No significant drug-related changes associated with clinical symptoms were found in four groups using ophthalmoscopy and clinical examination.

According to our findings, body weights of the rats in administration groups were significantly lower in week 6 compared to control ones. Nonetheless, no significant difference in the body weight and average feed conversion efficiency were found between the administration groups and control group during the following 90-day period. It is generally accepted that the reduction of body weight gains or losses 10% more than the mean control value is defined as toxicological significance (34). Therefore, our study suggests that the EFE administration had no compound-related effects on body weight changes in rats.

As for the hematological examination, the mean red blood cell volume (MCV) in the low-dose group was higher than the control one. The MCV increase in the EFE-1 group was not dose-related because the effect was not compounded at the EFE-2 and EFE-3 groups. Additionally, compared with the control group, the mean hemoglobin content (MCH) and mean hemoglobin concentration (MCHC) in the EFE-3 group were found increased by 3.07% and 1.82%, respectively. A previous study suggested that the increase of MCV, MCH, as well as MCHC was correlated with the changes of blood viscosity and RBC deformability (35). However, these statistical differences were not considered adverse or related to the consumption of EFE because these values were comparable between the one EFE group and control group. The lymphocyte count (LY) and the mean platelet volume (MPV) were decreased by 18.96% and 3.32%, respectively.

In terms of chemical and biological examination, total cholesterol (T-CHO) and triglyceride (TG) decreased by 20.67% and 46.15% in the group of EFE-3 compared with the control one, which may result from the prolonged fasting in the rat before treatment, inducing the excessive binding of triglycerides to proteins. However, the values of TG detected were too low to be statistically analyzed under certain circumstances, which might obscure the investigative process.

Although there were several statistically significant differences between the drug groups and control group, no significant dose-dependent relationship effects were discovered in these abnormal indicators. Consequently, the changes of hematology and biochemical indicators in rats should not be considered as the compound-related effects and was not considered biologically significant.

In the analysis of organ weight and histopathological, though significant differences in absolute weights were discovered in lung and spleen, no obvious lesions were found in further histopathological examination and these changes could be considered to be incidental.

Overall, compared with the control group, we discovered a decrease in the body weight, T-CHO, as well as TG, and an increase in the MCV and MCH along with MCHC in the EFE group. However, these alterations were not dose-related, and no significant correlations were found with the EFE. We attribute this phenomenon to some extraneous factors as well as biological variations arising from inter- and intra-animal components. In addition, other physiological factors including age, sex, restraint, diet, and circadian may also have the effects on the overall variation. The results of our research confirm that there are no adverse effects due to the administration of EFE in traditional medicine.

## 5. Conclusion

To the best of our knowledge, this is the first study on the safety assessment of EFE. In the present study, the results suggest that EFE on prolonged administration may cause a slight change in body weight and hematological examination but could harbor no significant toxicological effect. Therefore, our study provides some evidence on the safety of the ethanol extract of *Eupatorium japonicum Thunb* and *Foeniculum vulgare* for potential clinical application. In addition, further studies of EFE are still necessary to assess its oral safety in patients.

## Figures and Tables

**Table 1:** Body weight, food consumption, and food conversion efficiency of rats fed with EFE for 90 days.

	EFE-1	EFE-2	EFE-3	Control
Day 0	247.30 ± 11.19^a^	246.75 ± 16.50	238.30 ± 15.13	242.30 ± 13.48
Day 4	270.95 ± 17.14	278.25 ± 18.35	274.55 ± 16.76	277.90 ± 18.10
Day 8	298.35 ± 16.73	295.80 ± 15.97	295.10 ± 18.61	302.35 ± 15.81
Day 12	311.30 ± 16.59	317.95 ± 16.47	314.00 ± 18.31	317.65 ± 13.49
Day 16	336.35 ± 18.99	335.45 ± 19.85	339.20 ± 18.88	346.50 ± 18.07
Day 20	360.20 ± 20.16	369.70 ± 20.77	356.85 ± 20.80	365.45 ± 16.13
Day 24	376.05 ± 20.35	383.55 ± 24.70	370.85 ± 20.48	377.40 ± 17.82
Day 28	381.05 ± 31.27	409.45 ± 29.01	388.55 ± 20.42	396.80 ± 16.49
Day 32	396.65 ± 34.42	429.10 ± 28.58	405.50 ± 22.53	417.05 ± 21.23
Day 36	410.25 ± 36.33	450.10 ± 30.37	424.85 ± 24.47	437.75 ± 25.02
Day 40	432.60 ± 26.74^∗^	458.20 ± 35.39	445.00 ± 26.38	460.50 ± 26.47
Day 44	459.15 ± 30.61	473.65 ± 39.06	456.50 ± 28.38	453.25 ± 21.02
Day 48	471.95 ± 32.48	491.50 ± 38.86	470.95 ± 30.23	471.85 ± 24.53
Day 52	482.60 ± 33.41	499.55 ± 40.19	477.15 ± 29.88	482.75 ± 27.98
Day 56	489.00 ± 30.45	515.00 ± 43.12	486.25 ± 26.44	496.95 ± 29.29
Day 60	499.05 ± 33.10	520.75 ± 43.49	492.05 ± 27.81	505.70 ± 30.78
Day 64	509.45 ± 31.20	530.50 ± 45.37	503.35 ± 29.72	511.85 ± 34.32
Day 68	513.35 ± 35.32	534.95 ± 47.58	512.05 ± 31.34	515.75 ± 33.98
Day 72	526.30 ± 38.29	549.50 ± 48.06	519.80 ± 33.01	527.90 ± 35.86
Day 76	526.95 ± 40.44	549.20 ± 48.32	521.30 ± 38.99	533.95 ± 35.85
Day 80	533.40 ± 39.71	563.00 ± 49.26	533.35 ± 35.14	545.95 ± 41.58
Day 84	537.50 ± 39.42	576.60 ± 45.83	538.40 ± 32.89	553.45 ± 40.33
Day 88	542.50 ± 42.21	571.60 ± 51.51	539.78 ± 36.18	557.90 ± 38.09
Day 90	512.60 ± 37.70	563.30 ± 51.29	515.00 ± 35.48	535.75 ± 36.28
Total food consumption^b^	1587.3 ± 98.6	1923.7 ± 91.5	1685.6 ± 86.5	1807.5 ± 76.4
Average feed conversion efficiency (%)^c^	16.85 ± 0.98	16.51 ± 0.82	16.48 ± 0.69	16.41 ± 0.77

^a^Body weight means (g/rat ± SD), food consumed means (g/rat/day ± SD, values in parenthesis), and average food conversion efficiency [(g weight gain)/(g feed consumed) ± SD] of Sprague–Dawley rats (*n* = 5/dose) consuming different concentrations of EFE. ^b^Total food consumption: total food consumed since study initiation. ^c^Average feed conversion efficiency: total food consumption divided by total body weight gain. Values are significantly different (^∗^*P* < 0.05) as compared with the corresponding control group.

**Table 2:** Hematology in rats given EFE for 90 days.

	EFE-1	EFE-2	EFE-3	Control
WBC (×10^9^/L)	8.42 ± 3.57^a^	9.29 ± 3.32	6.13 ± 2.40	9.65 ± 5.04
Lymphocyte (%)	78.07 ± 4.59	79.34 ± 5.82	71.39 ± 10.11	78.41 ± 5.80
Monocyte (%)	5.26 ± 1.51	4.32 ± 1.37	6.05 ± 2.84	10.51 ± 17.47
Neutrophil (%)	14.72 ± 3.85	14.69 ± 5.28	20.31 ± 7.77	14.83 ± 5.20
Eosinophil (%)	1.93 ± 0.71	1.64 ± 0.86	2.24 ± 1.43	1.64 ± 0.36
Basophil (%)	0.02 ± 0.07	0.01 ± 0.03	0.01 ± 0.04	0.01 ± 0.03
RBC (×10^12^/L)	8.60 ± 0.47	8.58 ± 0.33	8.55 ± 0.72	8.65 ± 0.57
Hb (g/L)	155.67 ± 7.62	152.90 ± 5.30	152.88 ± 10.49	152.10 ± 6.95
HCT	45.06 ± 2.26	44.52 ± 1.40	43.50 ± 2.90	43.88 ± 1.86
MCV	52.44 ± 1.46^∗^	51.92 ± 1.58	51.63 ± 0.93	50.85 ± 1.70
MCH	18.12 ± 0.59	17.83 ± 0.53	18.14 ± 0.19^∗^	17.60 ± 0.65
MCHC	345.44 ± 3.17	343.60 ± 2.46	351.38 ± 3.70^∗^	346.10 ± 5.49
CV RDW	14.98 ± 0.41	15.04 ± 0.51	15.26 ± 0.44	15.33 ± 0.48
LY	6.68 ± 3.08	7.46 ± 2.94	4.53 ± 2.21^∗^	9.50 ± 5.59
MO	0.46 ± 0.27	0.43 ± 0.25	0.36 ± 0.22	0.49 ± 0.24
NE	1.14 ± 0.32	1.26 ± 0.35	1.14 ± 0.30	1.33 ± 0.52
EO	0.15 ± 0.05	0.14 ± 0.05	0.11 ± 0.05	0.16 ± 0.09
LT	869.00 ± 79.52	883.10 ± 71.83	884.88 ± 128.70	954.60 ± 117.13
MPV	6.46 ± 0.21	6.49 ± 0.30	6.41 ± 0.22^∗^	6.63 ± 0.19

^a^Values are means ± standard deviations. Values are significantly different (^∗^*P* < 0.05) as compared with the corresponding control group.

**Table 3:** Serum biochemistry in rats given EFE for 90 days.

	EFE-1	EFE-2	EFE-3	Control
TP (g/L)	56.28 ± 2.68^a^	53.29 ± 1.64	57.06 ± 1.70	55.15 ± 3.51
ALB(g/L)	37.91 ± 1.99	35.31 ± 1.63	39.09 ± 1.51	37.13 ± 2.30
ALT(U/L)	33.56 ± 7.04	34.60 ± 7.47	29.00 ± 6.61	30.90 ± 6.24
AST(U/L)	101.44 ± 23.48	85.40 ± 14.78	110.63 ± 35.36	87.20 ± 11.77
BUN (mmol/L)	6.52 ± 1.00	4.90 ± 0.64	4.89 ± 0.71	5.49 ± 2.07
CERA(*μ*mol/L)	45.22 ± 5.89	36.50 ± 6.28	39.38 ± 10.20	41.10 ± 12.13
T-CHO (mmol/L)	1.68 ± 0.41	1.57 ± 0.25	1.42 ± 0.30^∗^	1.79 ± 0.29
TG (mmol/L)	0.81 ± 0.27	1.25 ± 0.47	0.42 ± 0.20^∗^	0.78 ± 0.39

^a^Values are means ± standard deviations. Values are significantly different (^∗^*P* < 0.05) as compared with the corresponding control group.

**Table 4:** Organ weights in rats given EFE for 90 days.

Item	EFE-1	EFE-2	EFE-3	Control
Absolute organ weight (g)				
Heart	1.383 ± 0.169^a^	1.418 ± 0.155	1.309 ± 0.095	1.383 ± 0.097
Lung	1.723 ± 0.133	1.826 ± 0.188	1.573 ± 0.057^∗^	1.690 ± 0.116
Liver	14.684 ± 1.676	16.280 ± 2.129	13.423 ± 0.980	13.634 ± 1.385
Spleen	0.863 ± 0.153	0.953 ± 0.187^∗^	0.734 ± 0.120	0.798 ± 0.071
Kidney	3.199 ± 0.257	3.295 ± 0.362	3.139 ± 0.355	3.036 ± 0.196
Brain	2.137 ± 0.064	2.128 ± 0.116	2.045 ± 0.149	2.059 ± 0.138
Thymus	0.369 ± 0.094	0.362 ± 0.068	0.396 ± 0.093	0.340 ± 0.063
Adrenal gland	0.051 ± 0.017	0.052 ± 0.009	0.046 ± 0.013	0.055 ± 0.013
Testes	3.712 ± 0.212	3.984 ± 1.014	3.393 ± 0.322	3.507 ± 0.197
Epididymis	1.297 ± 0.098	1.308 ± 0.306	1.180 ± 0.149	1.135 ± 0.274
Relative organ weight (%)				
Heart	0.25 ± 0.07	0.27 ± 0.03	0.24 ± 0.05	0.25 ± 0.03
Lung	0.35 ± 0.05	0.32 ± 0.02	0.29 ± 0.06	0.31 ± 0.05
Liver	2.86 ± 0.21	2.79 ± 0.28	2.63 ± 0.19	2.59 ± 0.26
Spleen	0.19 ± 0.08	0.22 ± 0.03	0.17 ± 0.04	0.17 ± 0.08
Kidney	0.63 ± 0.07	0.67 ± 0.09	0.58 ± 0.03	0.59 ± 0.04
Brain	0.47 ± 0.05	0.41 ± 0.03	0.39 ± 0.06	0.44 ± 0.05
Thymus	0.07 ± 0.008	0.06 ± 0.005	0.06 ± 0.007	0.07 ± 0.005
Adrenal gland	0.009 ± 0.0008	0.010 ± 0.0009	0.009 ± 0.0005	0.009 ± 0.0006
Testes	0.77 ± 0.08	0.69 ± 0.04	0.76 ± 0.05	0.73 ± 0.04
Epididymis	0.28 ± 0.06	0.26 ± 0.02	0.25 ± 0.04	0.26 ± 0.07

^a^Values are means ± standard deviations. Values are significantly different (^∗^*P* < 0.05) as compared with the corresponding control group.

## Data Availability

All data generated or analyzed during this study are included in this article.
